# Prefrontal and hippocampal microstructural gray matter following cognitive training under moderate hypoxia in mood disorders: a randomized controlled trial

**DOI:** 10.3389/fnins.2026.1798024

**Published:** 2026-04-08

**Authors:** Kristian H. R. Jensen, Ida P. Østergaard, Viktoria Damgaard, Johanna M. Schandorff, Julian Macoveanu, Cyril Pernet, Hannah W. Julsgart, Annette Johansen, Kristoffer Brendstrup-Brix, Lars V. Kessing, Martin B. Jørgensen, Hannelore Ehrenreich, Gitte M. Knudsen, Kamilla Miskowiak

**Affiliations:** 1 Neurobiology Research Unit, Rigshospitalet, Copenhagen, Denmark; 2 Copenhagen Affective Disorders Research Center (CADIC), Mental Health Center Copenhagen, Copenhagen, Denmark; 3 The Neurocognition and Emotion Across Disorders of the Brain (NEAD) Centre, Psychiatric Centre Copenhagen, Copenhagen, Denmark; 4 Department of Psychology, University of Copenhagen, Copenhagen, Denmark; 5 Department of Neurology, Copenhagen University Hospital, Rigshospitalet, Copenhagen, Denmark; 6 Department of Clinical Medicine, Faculty of Health and Medical Sciences, University of Copenhagen, Copenhagen, Denmark; 7 Experimental Medicine, Central Institute of Mental Health (CIMH), Mannheim, Germany

**Keywords:** altitude, cognition, DWI, hypoxia, mood disorders, neuroplasticity

## Abstract

**Background:**

Cognitive impairment persists during partial or full remission in 50–70% of individuals with mood disorders and impacts daily functioning and clinical prognosis. Preclinical evidence suggests that extended exposure to moderate hypoxia, combined with motor-cognitive learning, may elevate neuroplasticity and improve cognition. In these individuals with remitted mood disorders, we found that cognitive training under repeated moderate normobaric hypoxia improved executive function, and here investigate neurobiological mechanisms.

**Methods:**

Participants with major depressive disorder (MDD) or bipolar disorder (BD) in partial or full remission were randomized to 3 weeks of 3.5-h daily normobaric hypoxia (12% O_2_) combined with cognitive training five to 6 days per week or treatment-as-usual (TAU). Participants were assessed with cognitive tests and diffusion-weighted MRI at baseline and 1 month after treatment completion (week 8) as part of the ALTIBRAIN trial (ClinicalTrials.gov: NCT06121206). Prefrontal and hippocampal gray matter microstructure were modelled with Neurite Orientation Dispersion and Density Imaging (NODDI).

**Results:**

Fifty-seven participants (mean age 39 years, SD: 13, 70% female) with baseline MRI data were included. No significant effects of hypoxia-cognition training vs. TAU on neurite density index (NDI) or orientation dispersion index (ODI) were observed in either the prefrontal cortex or hippocampus (all p-FDR ≥ 0.832). No significant associations were observed between microstructural changes and changes in cognitive function in either region (all p-FDR ≥ 0.721). At baseline, microstructure in both regions was not associated with executive function or global cognition (all *p* > 0.40).

**Conclusion:**

The absence of detectable microstructural changes, despite selective improvements in executive function, indicates that NODDI-derived metrics did not capture structural correlates of the cognitive response to hypoxia-cognition training. Whether this reflects functional neural mechanisms, measurement insensitivity, or the timing of the single follow-up assessment remains to be determined. Future studies should incorporate multiple imaging time points to capture the dynamic trajectories of putative microstructural brain changes.

## 1 Introduction

Cognitive impairments, including difficulties with memory, concentration, and planning, are common in major depressive disorder (MDD) and bipolar disorder (BD) and critically influence functional outcomes such as quality of life, employment, and vulnerability to relapse, and patients also often report recovery of cognitive functions as a key treatment goal (Withall et al., 2008; Lam et al., 2014). However, cognitive deficits may persist in these mood disorders during remission and worsen with recurrent episodes (Demyttenaere et al., 2015; Chevance et al., 2020), representing a core feature rather than merely a consequence of mood symptoms. Cognitive dysfunction drives substantial functional disability across multiple domains: it impairs workplace productivity and contributes to high unemployment rates (Simon, 2003; McIntyre et al., 2015), whilst also affecting essential daily activities (Tse et al., 2014). Moreover, cognitive impairments may limit response to both pharmacological (Krueger et al., 2005) and psychological interventions (Wild and Gur, 2008; Tanev et al., 2020). These functional limitations translate into considerable socioeconomic costs (GBD 2019 Mental Disorders Collaborators, 2022).

Numerous randomised controlled trials (RCTs) have investigated possible pro-cognitive interventions. Most pharmacological and neurostimulation interventions have shown little or no benefit, and cognitive remediation programmes have produced overall small-to-moderate effects on cognition (Miskowiak et al., 2022; Woolf et al., 2022; Samamé et al., 2023). Given the limited efficacy of monotherapies, multimodal interventions may be needed to achieve robust, enduring improvements in cognition (Miskowiak et al., 2022). Converging evidence from clinical, preclinical, and *in vitro* studies suggests that cognitive impairments in mood disorders originate from deficits in neuroplasticity, characterised by reduced synaptic density and impaired rewiring of neural connections in response to environmental challenges (Price and Duman, 2020). Neurite Orientation Dispersion and Density Imaging (NODDI) uses diffusion-weighted MRI to model neurite density index (NDI), orientation dispersion index (ODI), and free water fraction in gray matter (Zhang et al., 2012). Cross-sectional studies have shown lower NDI in prefrontal regions in mood disorders (Nazeri et al., 2017; Ota et al., 2019; Sarrazin et al., 2019), and lower cortical NDI has been found in two studies to correlate with cognitive impairment in individuals with mood disorders (Nazeri et al., 2017; Nagai et al., 2025) and in two studies in healthy young and elderly populations (Gozdas et al., 2021; Brendstrup-Brix et al., 2024).

These microstructural changes may reflect the synaptic pathology observed in preclinical studies, where chronic stress and inflammation reduce dendritic spine density and complexity in the prefrontal cortex (PFC) and hippocampus (HPC) (Liao et al., 2025). At a molecular level, rodent and post-mortem studies of mood disorders have documented a reduction in neuroprotective factors, such as brain-derived neurotrophic factor (BDNF) (Price and Duman, 2020), with some human studies indicating that higher BDNF levels correlate with better cognitive function in mood disorders (Mora et al., 2019; Accardo et al., 2022; Çelebi et al., 2025). Notably, patients who improve with electroconvulsive therapy show increased NDI, providing evidence that symptom improvement involves neuroplasticity (Berre et al., 2025). Novel multimodal treatment strategies that directly target these underlying deficits in neuroplasticity and associated microstructural brain abnormalities are, therefore, a promising avenue for improving cognition across psychiatric conditions.

Controlled hypoxic exposure is an innovative treatment approach for enhancing neuroplasticity and improving cognition. Neurobiological responses to hypoxia are evolutionarily conserved and act as protective mechanisms that maintain central nervous system integrity during reduced oxygen levels, such as at high altitudes (Kaelin and Ratcliffe, 2008; Semenza, 2012). At the core of this adaptive response is hypoxia-inducible factor (HIF), which regulates cellular adaptation to hypoxia by increasing the expression of key proteins involved in cognitive function, including erythropoietin (EPO), and vascular endothelial growth factor (VEGF) (Kaelin and Ratcliffe, 2008; Semenza, 2012; Ehrenreich et al., 2023; Wang et al., 2025). High-altitude training, used by athletes to gain a physical edge (Mujika et al., 2019), may similarly be leveraged for ‘mental athletics’ by enhancing neuroplasticity and cognitive reserve (Burtscher et al., 2024). Indeed, repeated exposure to 10–16% O₂ (≈5,800 to 2,200 m altitude) for as little as half an hour has demonstrated neuroprotective effects and possible cognitive benefits in a systematic review of 56 preclinical and clinical studies (Damgaard et al., 2023).

Some studies suggest these effects are amplified when hypoxia is combined with motor-cognitive training, potentially through synergistic effects on neuroplasticity (Wakhloo et al., 2020; Ehrenreich et al., 2023), although conclusions are limited by small-scale trials and a high risk of bias across previous human studies (Damgaard et al., 2023). Yet, only a pilot study has investigated the putative pro-cognitive effects of hypoxia in a psychiatric population. They found that hypoxia combined with motor-cognitive training was well-tolerated across mood and autism spectrum disorders (Mennen et al., 2024), encouraging further studies.

We recently conducted an outcome-assessor-blinded randomized controlled trial (ALTIBRAIN) demonstrating that 3 weeks of daily 3.5-h of normobaric hypoxia (12% O₂, ≈4,400 m altitude) with cognitive training improved executive function (secondary trial outcome) in cognitively impaired individuals with mood disorders (Miskowiak et al., 2024). This intervention also increased neural efficiency during working memory 1 month post-treatment, as indicated by greater deactivation in the dorsal prefrontal cortex and the occipito-parietal cortex.^1^ To examine whether these executive function and neural activity changes were accompanied by any detectable changes in tissue microstructure, we investigated gray matter microstructural changes at 1 month after treatment completion using NODDI, which provides model-derived, indirect estimates of neurite volume fraction (NDI) and orientation dispersion (ODI). While these metrics cannot directly measure HIF signalling, molecular neuroplasticity cascades, or synaptic remodelling, they may reflect macroscopic tissue-level consequences of such processes if they occur at sufficient magnitude and persist to the measurement timepoint.

Based on the above, we hypothesised that H-CT would increase gray matter NDI and ODI in the PFC and HPC, consistent with NODDI model estimates of greater neurite volume fraction and orientation dispersion, which may indirectly reflect neurite density and branching complexity. We also explored microstructural changes specifically in the right rostral middle frontal cortex, a key region for executive functions and associated with blood-oxygen-level-dependent (BOLD) signal changes during working memory tasks following EPO treatment (Miskowiak et al., 2016). Lastly, we investigated whether any such changes correlated with changes in executive function, as well as the baseline correlation between NDI and ODI and cognitive function.

## 2 Methods

### 2.1 Study design

This sub-study includes data from 57 participants with MDD and BD from the larger ALTIBRAIN study who had baseline MRI scans; for the full study protocol, see (Miskowiak et al., 2024). The study was approved by the Committee on Health Research Ethics in the Capital Region of Denmark (protocol number: H-22028111), the Danish Data Protection Agency (protocol number: P-2022-354), and pre-registered at ClinicalTrials.gov (NCT06121206). All participants were informed about the study procedures and provided written informed consent.

### 2.2 Participants

Participants aged 18–65 with a primary diagnosis of MDD or BD in full or partial remission were recruited through the Mental Health Services in the Capital Region of Denmark, consulting psychiatrists, and advertisements on relevant websites between April 2023 and April 2025. Data collection was completed in August 2025. The ICD-10 diagnosis and full or partial remission status were confirmed using the semi-structured Schedules for Clinical Assessment in Neuropsychiatry (SCAN) interview and a score ≤14 on both the Hamilton Depression Rating Scale (HDRS) and Young Mania Rating Scale (YMRS). The participants also had to have objective or subjective cognitive impairments, defined as ≥0.5 SD below the age- and education-adjusted Danish norm on at least two of the five subtests on the Screen for Cognitive Impairment in Psychiatry (SCIP) (Ott et al., 2020) or ≥14 total score on the Cognitive Complaints in Bipolar Disorder Rating Assessment (COBRA) (Rosa et al., 2013). Functional Assessment Short Test (FAST) was also performed.

Exclusion criteria were organic mental disorders, alcohol or substance abuse, or schizophrenia spectrum disorders (ICD-10 codes F00-F29), a history of neurological disorders (including dementia, serious head trauma, and severe migraines), and daily use of ≥22.5 mg oxazepam or equivalent.

To ensure cardiovascular safety, BMI > 30 or any current or past severe medical conditions such as heart disease, lung disease, cancer, diabetes, renal failure, untreated or poorly controlled hypertension, thromboembolic events, or a first-degree relative who experienced a thromboembolic event before the age of 60, pregnancy or breastfeeding, attempting pregnancy, current intake of iron supplements, regular use of nicotine products or a history of altitude sickness were excluded. For MRI, claustrophobia and internal electronic or metal objects were contraindications.

### 2.3 Treatment groups

#### 2.3.1 Active treatment (H-CT)

The 3 weeks of active treatment consisted of 3.5-h sessions, 5–6 days per week, combining continuous moderate normobaric hypoxia with cognitive training (H-CT). Normobaric air with 12% O_2_ (equivalent to an altitude of ~4,400 m) was delivered into a 20 m^3^ room. The initial 16% O_2_ (~2,200 m, similar to airplane cabin level) was lowered to 12% over approximately 30 min, except for the first session, which used a 2-h progressive lead-in to minimize altitude sickness. Participants, seated at desks, used iPads for designated cognitive training with the validated, web-based Happy Neuron Pro (Danish version: 2024), designed around neuroplasticity principles.

Sessions included scheduled breaks, restroom breaks, and treadmill walking (3 × 10 min) to maintain comfort and prevent adverse sedentary effects. Blood oxygen saturation (SpO₂) and pulse rate were continuously monitored by an oximeter, with values displayed only outside the room for blinding. Medical doctors were on call for emergencies, including altitude sickness.

#### 2.3.2 Treatment-as-usual (TAU)

The treatment-as-usual (TAU) group continued their standard clinical care for mood disorders, as did the active intervention group. The TAU group also underwent all study assessments, including neurocognitive testing and MRI, in parallel with the active group. Upon completing the final assessment, TAU participants were offered the active intervention. A waitlist control group rather than an active control group was used to sustain motivation and minimise attrition in this time-demanding study.

### 2.4 Cognitive assessments

Participants were tested with 12 cognitive tests covering five domains, i.e., verbal learning and memory, executive functions, working memory, processing speed, and attention (for a list of specific tests, see Supplementary Table 1) at baseline and 1 month after treatment completion (approximately at week 8). Treatment effects on cognitive outcomes are the focus of a separate report (Schandorff et al., in preparation). Participants’ verbal IQ was evaluated using the Danish Adult Reading Test (Nelson and O’Connell, 1978), which was only assessed at baseline.

Composite domain scores were computed by z-transforming and averaging the raw scores using the baseline means and standard deviations from the total sample. Where necessary, variables were reversed to ensure that lower scores consistently reflected greater impairment.

The present study used cognitive change scores to examine brain-cognition associations. Here, the primary outcome of interest was the executive functions domain score (from the Trail Making Test—B, One Touch Stockings of Cambridge ‘mean choices to correct’ (CANTAB), Verbal fluency (total on letters ‘S’ and ‘D’), and the Wisconsin Card Sorting Task ‘Perseverative errors’). A secondary measure, global cognition composite score’, was also computed by averaging all five domain scores.

### 2.5 MRI acquisition

MRI was acquired at baseline and approximately at week 8, i.e., month after treatment completion. MRI was acquired on a 3-Tesla Magnetom Prisma (Siemens) scanner using a 32-channel head coil. T1-weighted (T1w) images were acquired using a MPRAGE-sequence in the sagittal plane (TR 2000 ms, TE 2.58 ms, 8° flip angle; 0.9 mm slice thickness, field of view (FOV) read 230 mm, base resolution 256). For diffusion-weighted imaging (DWI), a multishell protocol was acquired along 32 non-collinear directions at *b*-values (1,000, 2000 s/mm^2^), and 6 B₀ images were acquired without diffusion gradients. The shells were obtained using a multishot echo-planar spin-echo (EPSE) sequence (TR 3000 ms, TE 58 ms, Base Resolution 92, 2.5 mm slice thickness, FOV read 230 mm, simultaneous multislice factor = 2, phase partial Fourier = 7/8 with 50 slices acquired in ascending order per slice group). Gradient-echo (GRE) field maps (TR = 444 ms, TE = 4.92 ms, flip angle = 60°, 3 mm isotropic, SENSE acceleration, Phase-Encoding direction = j-) were acquired and used for B₀ correction of the subsequent functional runs.

### 2.6 NODDI estimation

Diffusion-weighted imaging (DWI) data were preprocessed using QSIprep 0.16.1 (Cieslak et al., 2021). T1-weighted images were processed using Advanced Normalization Tools (ANTs), including N4 bias field correction and skull-stripping via antsBrainExtraction. Spatial normalisation to the ICBM 152 template was performed through nonlinear registration, followed by tissue segmentation into cerebrospinal fluid, white matter, and gray matter using FSL’s FAST.

DWI preprocessing included MP-PCA denoising (MRtrix3), B1 field inhomogeneity correction, and intensity normalisation across B₀ images. Images with *b*-values <100 s/mm^2^ were treated as B₀ references. Head motion and eddy current corrections were applied using FSL’s pipeline with linear first- and second-order modelling to characterise spatial distortions. Outlier replacement was performed for slices that deviated by >4 standard deviations from predictions, with imputed values replacing aberrant data.

NODDI reconstruction was performed with QSIrecon 1.1.1 using the Accelerated Microstructure Imaging via Convex Optimization (AMICO) implementation (Daducci et al., 2015). NODDI was used to estimate microstructural properties, deriving the neurite density index (NDI; intracellular volume fraction) and orientation dispersion index (ODI). NDI and ODI maps were normalised to the standard Montreal Neurological Institute (MNI) template. Within each region of interest (ROI), regional metrics were summarised using QSIrecon’s tissue-weighted means, computed by weighting voxel-wise NDI and ODI values by the NODDI-derived tissue fraction (TF = 1 − free water fraction), thereby reducing bias arising from CSF partial-volume effects (Parker et al., 2021).

Prefrontal cortex and hippocampal regions were segmented using FastSurfer from the QSIprep’ed. T1w images and defined according to the Desikan-Killiany-Tourville (DKT) atlas. Mean NDI and ODI values were extracted from these regions for statistical analysis.

For the PFC, bilateral regions included the rostral and caudal anterior cingulate, rostral and caudal middle frontal, superior frontal, pars opercularis, pars triangularis, and pars orbitalis (left hemisphere codes: 1003, 1,027, 1,028, 1,018, 1,019, 1,020, 1,012, 1,014; right hemisphere codes: 2003, 2027, 2028, 2018, 2019, 2020, 2012, 2014).

### 2.7 Statistics

Analyses were performed in R (4.5.2) using the lmerTest and emmeans packages. Results are presented with [95% confidence intervals].

#### 2.7.1 Microstructural gray matter changes in the prefrontal cortex and hippocampus

We examined treatment effects on gray matter microstructure metrics, NDI, and ODI, in the PFC and HPC. Four linear mixed-effects models were fitted, i.e., one for each measure-region combination:


$$\begin{array}{l} \text{Microstructure metric}\sim \text{Time}\times \text{Treatment}+\mathrm{Age}+\\ \mathrm{Sex}+\text{Hemisphere}+(1\;|\;\text{Participant}) \end{array}$$

The TAU group served as the reference category. Participant was modelled as a random effect. The Time × Treatment interaction tested whether the change from baseline to follow-up differed between groups. Age was included as a covariate given established age-related trajectories in both hippocampal and prefrontal cortex NDI and ODI, with significant linear effects across the adult lifespan independent of volume changes (Nazeri et al., 2015).

To control for multiple comparisons, we applied the False Discovery Rate (FDR) correction using the Benjamini-Hochberg procedure with *α* = 0.05. Analyses followed intention-to-treat principles, with missing data handled using restricted maximum likelihood estimation.

As a descriptive check of baseline comparability, treatment-group differences in prefrontal and hippocampal NDI and ODI were estimated from the longitudinal mixed-effects models using baseline-estimated marginal means.

Exploratively, we also investigated changes specifically in the right rostral middle frontal cortex (DKT: 2027), which has been associated with blood-oxygen-level-dependent (BOLD) signal changes during working memory tasks in response to EPO treatment (Miskowiak et al., 2016).

As sensitivity analyses, we repeated the model using the first eigenvalue of the regional NDI and ODI distributions instead of the regional means to account for potential non-Gaussian and spatially heterogeneous regional distributions, particularly in larger regions (i.e., the PFC).

#### 2.7.2 Microstructural gray matter and cognition

We first examined cross-sectional associations between baseline brain microstructure and cognitive performance. NDI and ODI were averaged across both hemispheres in the PFC and HPC. Linear regression models were fitted for executive functions (primary outcome of interest) and global cognition (secondary outcome of interest) at baseline, with baseline microstructure as the predictor and age and sex included as covariates:


Cognitive function~Microstructure+Age+Sex

Next, we examined associations between microstructural changes and cognitive changes. NDI and ODI were averaged across both hemispheres in the PFC and HPC, as the primary analyses revealed bilateral changes in fMRI signal with no evidence of hemispheric lateralisation in response to H-CT (Schandorff et al., in preparation). We examined associations between NDI and ODI change and executive function and global cognition. For each combination, a linear regression model was fitted:


Cognitive change~Microstructure change+Treatment+Age+Sex

Because no significant treatment effects on the microstructure metrics were observed in the primary analyses, a treatment-by-microstructure interaction term was not included. FDR-corrected *p*-values are reported across the primary comparisons. Lastly, the analyses were repeated for the right rostral middle frontal cortex volume only for executive functions.

## 3 Results

The participants had a median [range] age of 34 [21–64] years old and 70% female (Table 1). Seven of 30 participants allocated to H-CT dropped out or were excluded after baseline assessments (CONSORT diagram in Supplementary Figure 1). One participant who completed treatment did not receive a follow-up DWI scan due to scheduling difficulties. Of the possible 18 days of H-CT, the median [IQR] completion was 15 [14, 15] days.

**Table 1:** Baseline demographic and clinical characteristics of participants.

Characteristic	Overall *n* = 57	H-CT *n* = 30	TAU *n* = 27
Demographics			
Female	40 (70%)	23 (77%)	17 (63%)
Age (years)	34 [28; 51]	34.5 [30.5; 49.5]	31 [26.5; 52.5]
Educational years	15.6 (2.5)	15.4 (2.3)	15.9 (2.7)
Drop-out	7 (12%)	7 (23%)	0 (0%)
Missing follow-up DWI	8 (14%)	8 (27%)	0 (0%)
Diagnosis			
MDD	33 (58%)	18 (60%)	15 (56%)
BD-II	15 (26%)	7 (23%)	8 (30%)
BD-I	9 (16%)	5 (17%)	4 (15%)
Illness duration (years)	16 [9; 26]	16.5 [12.5; 31]	13 [8; 25.5]
No. depressive episodes	5 [2; 10]	5 [3.25; 10]	4 [2; 12.5]
No. hypomanic episodes	0 [0; 2]	0 [0; 1.5]	0 [0; 2.75]
No. manic episodes	0 [0; 0]	0 [0; 0]	0 [0; 0]
No. hospitalisations	0 [0; 1]	0 [0; 1]	0 [0; 1.5]
Psychometrics			
HDRS	3 [1; 6]	3 [1; 6]	3 [2; 6.5]
YMRS	0 [0; 2]	0 [0; 1]	0 [0; 2]
FAST	16 [16; 23]	17 [11.25; 25]	14 [10; 20]
Verbal IQ	112 (6.4)	112 (6.0)	112 (7.1)
Psychotropics			
Unmedicated	20 (35%)	8 (27%)	6 (22%)
Antidepressants^a^	23 (40%)	12 (40%)	11 (41%)
Lamotrigine	15 (26%)	8 (27%)	7 (26%)
Lithium	14 (25%)	6 (20%)	8 (30%)
Central stimulants	4 (7%)	3 (10%)	1 (4%)
Per necessitate^b^	21 (37%)	10 (33%)	11 (41%)

Data presented as *n* (%), mean (standard deviation), or median [quartiles]. BD-I, bipolar disorder type 1; BD-II, bipolar disorder type 2; FAST, Functioning Assessment Short Test; HDRS, 17-item Hamilton depression rating scale; MDD, major depressive disorder; TAU, treatment as usual; YMRS, Young Mania Rating Scale. DWI, Diffusion-weighted image.

a SSRI, SNRI, and TCA.

b Anixolytics and sleep aids, i.e., benzodiazepines, melatonin, and low-dose Quetiapine.

**Figure 1:**
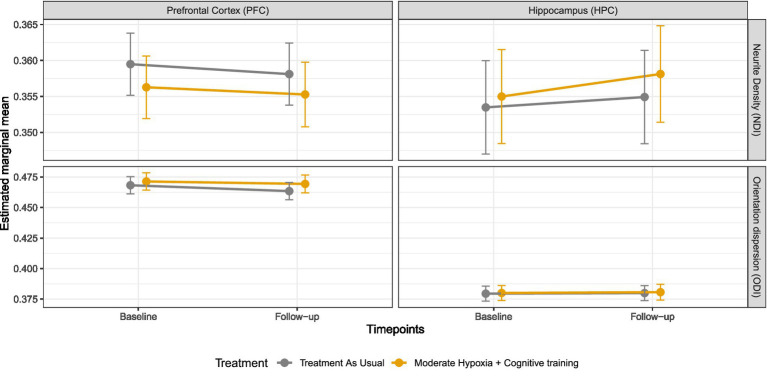
Microstructural gray matter changes in the prefrontal cortex and hippocampus. Estimated marginal means (±95% CI) for neurite density and orientation dispersion indexes across treatment groups and time points (the hypoxia with cognitive training group [H-CT] is shown in orange). No significant Time × Treatment interactions were observed in the hippocampus or prefrontal cortex, indicating no differential longitudinal change between groups.

Baseline estimates of prefrontal and hippocampal NDI and ODI showed minimal, non-significant differences between treatment groups (|Hedges’ *g*| = 0.06–0.59; raw differences ≤0.003; *p*-values ≥0.28, Figure 1), indicating comparable microstructure at baseline.

The H-CT group showed significant improvements in executive function, both immediately after the intervention (at week 4) and at the one-month follow-up assessment (at week 8), relative to TAU.

### 3.1 Microstructural gray matter changes following altitude-like hypoxia cognition training

Four linear mixed-effects models were fitted to examine treatment effects on the microstructure of the PFC and HPC (Figure 1). No significant Time × Treatment interactions were observed for NDI in either region [PFC: *β* (95% CI) = 0.0004 (−0.0027, 0.0034), p-FDR = 0.937; HPC: *β* = 0.0017 (−0.0024, 0.0058), p-FDR = 0.8832], indicating no differential change in neurite density over time between treatment groups. Similarly, no significant treatment effects were detected for ODI [PFC: *β* = 0.0028 (−0.0023, 0.0079), p-FDR = 0.832; HPC: *β* = 0.0028 (−0.0023, 0.0079), p-FDR = 0.832].

In sensitivity analyses, we repeated the treatment effect models using the first eigenvalue of the regional NDI and ODI distributions (rather than the regional mean) to account for potential regional heterogeneity. These analyses also showed no evidence of a treatment effect in either PFC or HPC (all p-values ≥ 0.307; all p-FDR ≥ 0.674).

Exploratory analyses in the right rostral middle frontal cortex also showed no significant Time × Treatment interactions were NDI [*β* = 0.0018 (−0.0055; 0.0090), *p* = 0.622] or ODI [*β* = 0.0056 (−0.0066, 0.0176), *p* = 0.371].

### 3.2 Associations between gray matter microstructure and cognition

**Figure 2:**
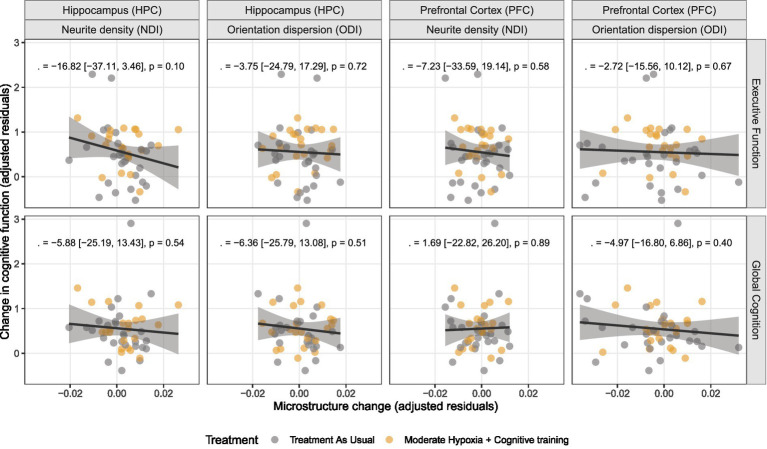
Microstructure and global cognitive performance. Scatter plots of the relationships between changes in brain microstructure in the hippocampus and prefrontal cortex (average across hemispheres) and changes in cognitive functions. Each point represents a participant, colored by treatment (gray = treatment as usual (TAU); orange = hypoxia + cognitive training). Solid dark gray lines indicate linear regression fits adjusted for treatment group, age, and sex, with shaded 95% confidence intervals.

At baseline, neither NDI nor ODI in any region was associated with executive function or global cognition (Supplementary Table 3, all *p* > 0.40). We assessed the relationship between microstructural changes in the bilateral PFC and HPC and changes in cognitive performance using linear regression models, adjusted for treatment group, age, and sex (Supplementary Table 2). The strongest estimated relation was observed between hippocampal NDI and executive function [*β* = −16.8, (−37.1, 3.46), *p* = 0.102, p-FDR = 0.814], although non-significant. No statistically significant associations were observed between cognition and changes in the PFC or HPC (Figure 2) or the right rostral middle frontal cortex (Supplementary Figure S2).

## 4 Discussion

This RCT investigated whether 3 weeks of normobaric hypoxia combined with cognitive training would induce gray matter microstructural changes in cognitively impaired patients with mood disorders. Contrary to our hypothesis, we observed no significant treatment effects on neurite density or orientation dispersion indices in the PFC or HPC, and no significant associations between microstructural changes and cognitive performance. At baseline, no association was observed between microstructural measures and cognition.

The lack of significant H-CT effects on prefrontal and hippocampal NDI and ODI was unexpected, given the preclinical evidence that hypoxia can increase neural plasticity (Damgaard et al., 2023; Ehrenreich et al., 2023). In the ALTIBRAIN trial we observed sustained improvements in executive functions (secondary outcome) at H-CT completion (approximately week 4) and 1 month follow-up (approximately week 8), but not in speed of complex cognitive processing (primary outcome), alongside neural changes using functional MRI at 1 month follow-up [i.e., greater deactivation in bilateral PFC during working memory tasks (Schandorff et al., in preparation)]. These functional changes, coupled with selective cognitive improvements in the absence of detectable microstructural alterations, indicate that H-CT enhances neural processing without producing gross changes in gray matter microstructure detectable by NODDI. Together, these findings suggest that NDI and ODI, as measured at 1 month post-intervention, did not change in response to 3 weeks of H-CT. Whether these metrics could serve as biomarkers of cognitive improvement following hypoxia with different timing or intensity protocols cannot be determined from the present data. However, preclinical evidence demonstrates that moderate hypoxia increases dendritic branching (Wakhloo et al., 2020). Therefore, several possible explanations for the present null NODDI findings warrant consideration.

First, the single follow-up time point at week 8, 1 month after treatment completion, may have been poorly timed to detect these microstructural changes. If initial synaptogenesis occurred during the three-week intervention, subsequent consolidation and pruning could have resolved any transient structural signal by the time of MRI assessment. This timing hypothesis is consistent with the improvements in executive functions observed here and in the companion paper (Schandorff et al., in preparation), which strengthened over time, suggesting ongoing functional consolidation rather than simple persistence of an acute effect. The ALTIBRAIN trial also includes concurrent synaptic density imaging using PET imaging at week 4, immediately after the three-week intervention (Miskowiak et al., 2024). Whether this earlier assessment reveals transient differences in synaptic density will be reported separately and may provide relevant, though not definitive, evidence bearing on this interpretation.

A second explanation is that the intervention was of insufficient intensity or duration to produce changes detectable by NODDI in this population. ECT induces hippocampal NDI increases within 2 weeks (Berre et al., 2025) and represents a substantially more potent neuroplastic stimulus. Age-related NODDI changes in healthy populations accumulate over decades (Nazeri et al., 2015). These observations suggest that 3 weeks of controlled hypoxia may be insufficient to produce measurable changes in neurite density or orientation dispersion.

Third, the neurobiological response to hypoxia may differ in a clinical sample with chronic mood disorders (mean illness duration 16 years), in whom pre-existing microstructural pathology and reduced neuroplastic reserve could limit the structural response, independent of any functional benefit. The absence of baseline microstructure-cognition associations is consistent with this, as previous studies reporting NODDI-cognition correlations examined samples with shorter illness durations (11 and 14 years) and younger mean ages (32 and 35 years) (Nazeri et al., 2017; Nagai et al., 2025), suggesting that microstructural-cognitive relationships may attenuate in more chronic presentations. The absence of baseline microstructure-cognition associations in the present cohort may reflect this, although it could equally reflect restricted variability, limited statistical power, or the cross-sectional nature of the baseline assessment, and should not be generalised beyond this sample. These explanations are not mutually exclusive and cannot be distinguished with the present data.

### 4.1 Strengths and limitations

Study strengths include the RCT design, repeated MRI scans of a relatively large initial sample, comprehensive cognitive assessment, tissue-weighted NODDI estimation (Parker et al., 2021), and rigorous statistical analyses with FDR correction. Yet, limitations warrant consideration.

The substantial attrition in H-CT (27% missing follow-up neuroimaging) reduced power. A sensitivity analyses indicated that the study had 80% power only to detect effects of Cohen’s d > 0.8. In contrast, all observed effect sizes were small (approx. d < 0.3), and their confidence intervals were narrowly centred around zero. For example, HPC NDI showed *β* = 0.0021 with a 95% CI [−0.0028, 0.0069], a range compatible only with very small effects in either direction. This pattern suggests that any true microstructural changes, if present, are minimal rather than masked solely by limited power. Critically, the single follow-up time point at 1 month after treatment completion may have missed transient microstructural changes occurring during or immediately after the three-week intervention. Lastly, the active condition combined hypoxia with cognitive training, and the waitlist TAU control does not permit disentangling the independent contributions of each component. Mechanistic interpretations regarding hypoxia-specific neuroplastic effects should therefore be considered speculative.

## 5 Conclusion

This RCT found no significant H-CT-related effects on gray matter microstructure in the prefrontal cortex or hippocampus 1 month after treatment completion. The dissociation between treatment-related cognitive improvements and the absence of structural changes suggests that NODDI-derived NDI and ODI, measured at 1 month post-treatment, did not reflect the neural substrate of the cognitive response to H-CT. Whether this reflects a genuine absence of structural plasticity, measurement insensitivity, or the timing of the single follow-up assessment cannot be determined from the current data. Future studies should incorporate multiple imaging timepoints and complementary methods to better characterise the temporal and structural dynamics of hypoxia-cognition training associated neuroplasticity.

## Data Availability

The original contributions presented in the study are included in the article/Supplementary material, further inquiries can be directed to the corresponding author.
