# Supplementary material for: Prefrontal and hippocampal microstructural gray matter following cognitive training under moderate hypoxia in mood disorders: a randomized controlled trial
Source: Front Neurosci. 2026 Apr 8;20:1798024. doi: 10.3389/fnins.2026.1798024 (PMC13099808; doi:10.3389/fnins.2026.1798024)

Supplementary Material

#### Supplementary Table S1: Cognitive domains and cognitive tests

Summary of neuropsychological measures used to assess the following cognitive domains: verbal learning and memory, executive functioning, working memory, processing speed, and attention. CANTAB: Cambridge Neuropsychological Test Automated Battery, RAVLT: Rey Auditory Verbal Learning Test, RBANS: Repeatable Battery for the Assessment of Neuropsychological Status, WAIS: Wechsler Adult Intelligence Scale.

| **Domains** | **Neuropsychological tests** |
| --- | --- |
| Executive  functions | - Trail Making Test – B - One Touch Stockings of Cambridge ‘mean choices to correct’ (CANTAB) - Verbal fluency (total on letters ‘S’ and ‘D’) - Wisconsin Card Sorting Task ‘Perseverative errors’ |
| Working  memory | - WAIS Letter-number sequencing - Spatial Working Memory ‘error’ (CANTAB) - Spatial Working Memory ‘strategy’ (CANTAB) |
| Processing  speed | - Trail Making Test – A - RBANS Coding |
| Attention | - Rapid Visual Processing ‘accuracy’ and ‘mean latency’ (CANTAB) - RBANS Digit span |
| Verbal learning  and memory | - RAVLT subtests (trial I-V total recall, immediate recall, and delayed recall) |

#### Supplementary Table S2. Changes in microstructure and changes in cognitive function

Associations between changes in regional brain microstructure and changes in cognitive performance. Estimates (β) and 95% confidence intervals were from linear regressions adjusted for age, sex, and treatment. Right rostral middle frontal (RMF) cortex analyses were exploratory and not FDR-corrected.

| **Region** | **Measure** | **Cognitive domain** | **β** | **95% CI** | **p-value** | **p-FDR** |
| --- | --- | --- | --- | --- | --- | --- |
| Bilateral  prefrontal cortex | NDI | Executive  function | −7.23 | [−33.6, 19.1] | 0.583 | 0.824 |
|  | ODI |  | −2.72 | [−15.6, 10.1] | 0.672 | 0.824 |
|  | NDI | Global  cognition | 1.69 | [−22.8, 26.2] | 0.890 | 0.890 |
|  | ODI |  | −4.97 | [−16.8, 6.86] | 0.402 | 0.824 |
| Bilateral  hippocampus | NDI | Executive  function | −16.8 | [−37.1, 3.46] | 0.102 | 0.816 |
|  | ODI |  | −3.75 | [−24.8, 17.3] | 0.721 | 0.824 |
|  | NDI | Global  cognition | −5.88 | [−25.2, 13.4] | 0.542 | 0.824 |
|  | ODI |  | −6.36 | [−25.8, 13.1] | 0.513 | 0.824 |
| Right  RMF cortex | NDI | Executive  function | 5.56 | [−8.04, 19.2] | 0.414 |  |
|  | ODI |  | −0.47 | [−8.58, 7.63] | 0.907 |  |

####

#### Supplementary Table S3. Microstructure and cognitive function at baseline

Associations between regional brain microstructure and cognitive function. Estimates (β) and 95% confidence intervals were from linear regressions adjusted for age and sex. Right rostral middle frontal (RMF) cortex analyses were exploratory and not FDR-corrected.

| **Region** | **Measure** | **Cognitive domain** | **β** | **95% CI** | **p-value** | **p-FDR** |
| --- | --- | --- | --- | --- | --- | --- |
| Bilateral  prefrontal cortex | NDI | Executive  function | 3.60 | [−15.0, 22.2] | 0.700 | 0.992 |
|  | ODI |  | 2.42 | [−9.12, 14.0] | 0.675 | 0.992 |
|  | NDI | Global  cognition | -0.12 | [−23.0, 22.8] | 0.992 | 0.992 |
|  | ODI |  | −0.50 | [−14.7, 13.7] | 0.944 | 0.992 |
| Bilateral  hippocampus | NDI | Executive  function | 0.85 | [−11.6, 13.2] | 0.892 | 0.992 |
|  | ODI |  | 2.63 | [−13.3, 18.6] | 0.742 | 0.992 |
|  | NDI | Global  cognition | −4.44 | [−19.6, 10.7] | 0.560 | 0.992 |
|  | ODI |  | −6.36 | [−25.8, 13.1] | 0.513 | 0.992 |
| Right  RMF cortex | NDI | Executive  function | 11.7 | [-11.3, 14.7] | 0.793 |  |
|  | ODI |  | 3.59 | [−4.93, 12.1] | 0.402 |  |

#### Supplementary Figure S1: CONSORT-diagram
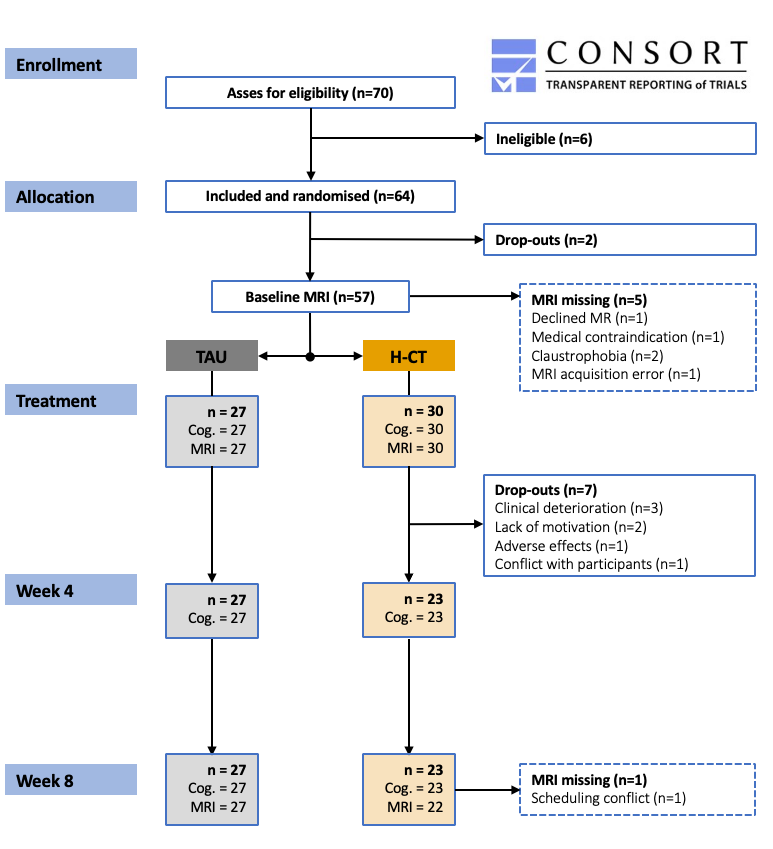


####

#### Supplementary Figure S2: Microstructure and global cognitive performance in the right RMF

Scatter plots illustrating the relationships between microstructure changes in the right rostral middle frontal (RMF) cortex. Each point represents a participant, coloured by treatment (gray = treatment as usual (TAU); orange = hypoxia + cognitive training). Solid dark gray lines indicate linear regression fits estimating the association between brain and cognitive change, adjusted for treatment group, with shaded 95% CI.


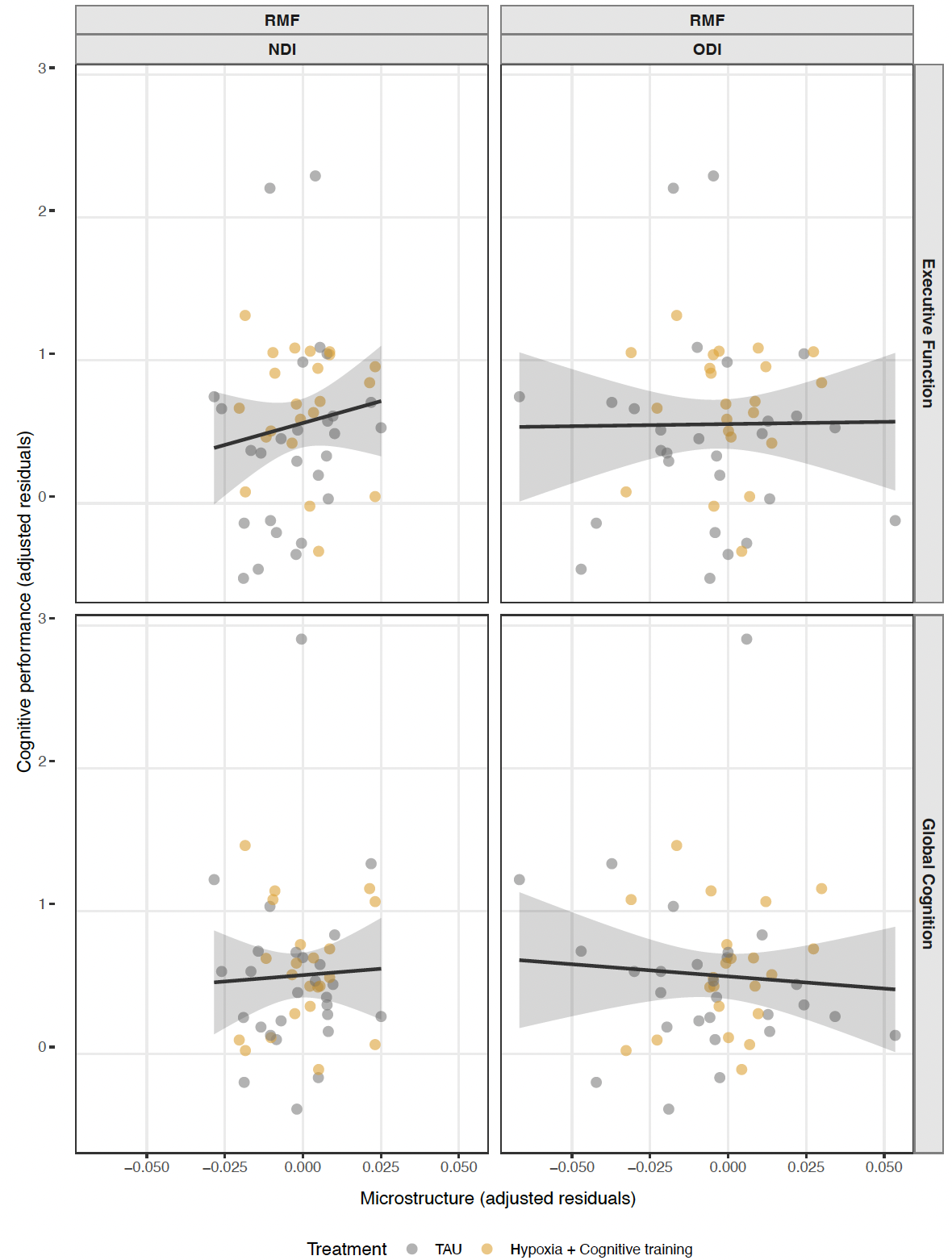

Supplement: Supplementary file 1 [file Data_Sheet_1.docx]
